# The protective effect of 17 β-estradiol on human uterosacral ligament fibroblasts from postmenopausal women with pelvic organ prolapse

**DOI:** 10.3389/fphys.2022.980843

**Published:** 2022-10-10

**Authors:** Ting Xie, Dan Guo, Tao Guo, Yapei Zhu, Fangyuan Li, Sumei Zhang, Jinghe Lang, Zhijing Sun

**Affiliations:** ^1^ Department of Medical Research Center, Peking Union Medical College Hospital, Chinese Academy of Medical Sciences & Peking Union Medical College, Beijing, China; ^2^ Department of Obstetrics and Gynecology, Peking Union Medical College Hospital, Chinese Academy of Medical Sciences & Peking Union Medical College, National Clinical Research Center for Obstetric & Gynecologic Diseases, Beijing, China

**Keywords:** pelvic organ prolapse, human uterosacral ligament fibroblasts, 17 β-estradiol, mechanical stress, PARP1

## Abstract

This study aims to explore the protective effects of 17 β-estradiol on the human uterosacral ligament fibroblasts (hUSLFs) under static or stretched conditions. The experiments were performed on hUSLFs derived from pelvic organ prolapse (POP) and non-POP patients. Fibroblasts were cultured after collagenase digestion and identified by morphological observation and immunocytochemical methods. 17 β-estradiol (10^−10^ M and 10^−9^ M) and mechanical stress induced by the FX-5000 T-cell stress loading system under a loading strain of 1/2 sin waveform uniaxial cyclic stress with a tensile strain of 20% and a frequency of 0.5 Hz were either or both applied on hUSLFs. Cell proliferation was measured by CCK8, and cell apoptosis and death were detected using Annexin V/7-AAD staining and flow cytometric analysis. We found that the fibroblasts growth rate of POP patients was significantly lower than controls. The cell apoptosis and death rate increased as the mechanical load intensifying. After 20% mechanical stretching for 24 h, the dead cell rate was higher in POP than control. Notably, 17 β-estradiol treatment reversed mechanical stress induced hUSLFs apoptosis and death in both POP and Control cells. The protein and mRNA levels of anti-apoptotic PARP1 (poly-ADP-ribose polymerase) and Bcl-2 were increased by estrogen treatment. Meanwhile, expression of estrogen receptor α, a target of Poly-ADP-Ribosylation of PARP1, was also enhanced by 17 β-estradiol under the mechanical load. In conclusion, estrogen application ameliorates the mechanical strain induced cell apoptosis and death in hUSLFs from POP patients. PARP1 might be involved in this protective process, providing novel insights into the mechanical biology of and possible therapies for POP.

## Introduction

Pelvic organ prolapses (POP) is commonly caused by over-laxity of the vaginal connective tissue or its supporting ligaments, which substantially affects the life quality of patients. POP includes herniation of the anterior, posterior, and apical vaginal compartments through the vaginal introitus according to the vaginal segment (Weintraub et al., 2020). As described in our previous studies, the prevalence of symptomatic POP was 9.6% in China, and the incidence of POP in women aged 50 years or older was reported to be nearly twice as high as women between the age of 20–29 (Pang et al., 2021). Although the etiology of POP is probably multifactorial, the advanced age and multiple vaginal deliveries are the major risk factors that contribute to weakening of the pelvic floor connective tissue (Vergeldt et al., 2015).

With increased life expectancy and the movement towards improved quality of life, not only the incidence of POP but also the prevalence of women seeking medical treatment for their symptoms increases, which will cause both medical and financial burdens. Aging, and specifically postmenopausal estrogen deficiency is a major cause for the prevalence of POP implicating the hypo estrogenic environment as a risk factor, because circulating levels of endogenous estrogen dip dramatically with menopause (Tinelli et al., 2010). The constituents for pelvic organ support including collagen, elastin, and fibroblasts are profoundly influenced by the estrogen levels and the expression of estrogen receptors. However, the influence of estrogen and selective estrogen receptor modulators (SERM) on the development of POP are still contradictory. Treatment with estradiol was shown to increase mRNA for collagen I and III in the vaginal connective tissues of rhesus macaque monkeys (Clark et al., 2005). Moreover, vaginal estrogen application increased thickness of the epithelium and muscularis of vaginal wall, enhanced synthesis of mature collagen, and decreased collagen degradative enzyme activity in the postmenopausal women with prolapse (Rahn et al., 2014). On the other hand, it was found that levormeloxifene (a kind of SERM) treatment led to a marked increase in the incidence of uterovaginal prolapse compared with placebo (7% vs. 2%, respectively) (Goldstein and Nanavati, 2002). Meanwhile, systemic hormone therapy may have a minor effect on pelvic organ support (Wasenda et al., 2017). Thus, we hope to evaluate the potential role of estrogen treatment in the fibroblast cells from the uterine ligament tissue of patients with or without severe POP.

Another main risk factor for POP is mechanical strain including vaginal delivery, parity, and the delivery of large infants, which increase intra-abdominal pressure and damage the muscles and fascia in the pelvic floor (Vergeldt et al., 2015). However, the underlying mechanisms of the effects of mechanical forces on POP is still under a veil. Fibroblasts constitute the majority of the ligaments cells (Al-Azzawi et al., 2003), which remodel extracellular matrix (ECM) in response to mechanical and biochemical stimuli to maintain tissue strength. A substrate stretching method is used to implement mechanical strain on fibroblasts *in vitro* to mimic the environment *in vivo*, and uniaxial cyclic stretching studies investigate the effects of mechanical loads on fibroblasts cultured on a two-dimensional substrate. It is reported that mechanical strain is associated with oxidative stress, decreased mitochondrial membrane potential, and increased apoptotic rate in human parametrial ligament fibroblasts (Hong et al., 2015a). The same research team also proved the mechanical forces induce mitochondrial injury, cytoskeletal alterations and increased cell senescence, resulting in decreased cell viability of pelvic fibroblasts (Ming et al., 2017). It is worth noting that the mechanical properties of fibroblasts from the vaginal wall connective tissues could also be improved by estrogen therapy, because this has been found to suppress excessive and abnormal remodeling of the fibroblasts and their cytoskeletons (Wang et al., 2015).

Considering the role that estrogen play in maintaining the integrity of pelvic floor connective tissues, in-depth studies are needed on the performance of exogenous estrogen administration in POP prevention. We speculate that the control and POP fibroblasts from the uterosacral ligament tissues will present different behaviors when uniaxial cyclic mechanical stretching acts on them. Fibroblast is the major cell component in ligaments producing collagen, elastic fibers and other ECM proteins, and will be used as the *in vitro* model for study in this research. The aim of this study was to investigate the efficacy of exogenous estrogen administration on human uterosacral ligament fibroblasts (hUSLFs) under static or mechanical stretching and the underlying mechanisms.

## Materials and methods

### Participants

The study protocol was approved by the Peking Union Medical College Hospital ethics committee. All subjects gave their written informed consent to participate in the study. Fresh human uterosacral ligament tissues were obtained from ten participants: five women (aged 55–68 years) with stage III or IV POP according to the Pelvic Organ Prolapse Quantification (POP-Q) System and underwent total vaginal hysterectomy, and five control women (aged 51–60 years) patients who suffered from benign gynaecological diseases (such as cervical intra-epithelial neoplasia and non-functional ovarian benign cysts) and underwent laparoscopic-assisted vaginal hysterectomy. We excluded women with urinary incontinence; uterine leiomyoma, adenomyosis, endometriosis or other estrogen related diseases; chronic pelvic inflammation; malignant tumors; or collagen deficiency syndrome and those who underwent previous pelvic surgery.

### Primary cell culture

The fresh uterosacral ligament tissues were collected from the posterior attachment to the cervix during surgery. After excision from the donor, the tissues were immediately washed twice with Dulbecco’s phosphate-buffered saline (DPBS) with 1% penicillin/streptomycin (P/S, Gibco, United States). Then the tissues were minced into 1 mm^3^ cubes with sterile ophthalmic scissors and placed evenly on a 2.5 cm^2^ polystyrene petri dish (Corning, United States) with Phenol Red-free Dulbecco’s modified Eagle’s medium (DMEM, supplemented with 10% FBS and 1% P/S). The culture medium was changed every 2 days, and primary fibroblasts grown out from the cubes to spread across the culture medium for passage after about 7 days, the fibroblasts were sub-cultured until the cells reached 90% confluence. After identification as the fibroblasts, the cells at passage 3–7 were collected by trypsin digestion and used in the following experiments.

### Immunocytochemistry

To confirm the vaginal connective tissue fibroblastic origin, the cells were permeabilized with 0.1% Triton X-100 and then incubation with specific antibodies overnight at 4°C including anti-vimentin (1:200, ZSGB-Bio, China, the major intermediate filament in fibroblast cells), anti-fibroblast specific protein 1 (FSP-1) (1:200, ZSGB-Bio, China, a specific marker of fibroblasts), anti-α-smooth muscle actin (α-SMA) (1:200, ZSGB-Bio, China, a maker predominating within vascular smooth-muscle cells) and anti-cell keratin 5/6 (1:200, ZSGB-Bio, China, the intermediate filament in epithelial cells). DPBS was used as negative control in place of the primary antibody. The cells were then incubated with biotinylated secondary antibodies (1:4,000, ZSGB-Bio, China) for 2 h at room temperature. 3,3-Diaminobenzidine solution was then used to visualize the localization of the target proteins. Finally, the cells were counterstained with haematoxylin (ZSGB-Bio, China) for nuclear staining.

### Effect of 17 β-estradiol on cell proliferation assay

hUSLFs from POP patients and control at passage 3-7 were treated with different concentrations of 17 β-estradiol. The fibroblasts seeded at a density of 10^4^ cells/well in 96-well plates and cultured overnight, then the culture medium was replaced by FBS-free DMEM and the cells were cultured for 6 h. The medium was then replaced by DMEM (supplemented with 10% FBS and 1% P/S) containing different concentrations of 17 β-estradiol (0, 10^−11^, 10^−10^, 10^−9^ and 10^−8^ M, MCE, United States)). Different concentrations of 17 β-estradiol were freshly prepared by dissolving the 17 β-estradiol powder in absolute dimethyl sulfoxide and then serially diluted with medium. Each group of the experiment was done in triplicates. After culturing for 24 h, the cell viability was measured by Cell Counting Kit-8 (CCK-8) assay (Solarbio, China) at 24, 48, 72 and 96 h according to the manufacturer’s protocol.

### Loading of cyclic mechanical stretch and the administration of 17 β-estradiol

The hUSLFs at passage 3 - 5 were seeded at a density of 2 × 10^5^ per well on a UniFlex Culture Plate-Collagen Type I (Flexcell, United States), a 6-well plate that had an elastic basement membrane pretreated with type I collagen and incubated for 24 h. Then cells were subsequently starved in culture medium without FBS for 6 h and administered 10^−10^ M and 10^−9^ M 17 β-estradiol. Next, the cell plate was placed onto the strain loading plate of the FX-5000 T instrument (Flexcell, United States) under a loading strain of 1/2 sin waveform uniaxial cyclic stresswith a tensile strain of 20% tensile strain and a frequency of 0.5 Hz for 24 h. The experiments were repeated for three times.

### Quantitative real-time polymerase chain reaction

Total RNA was extracted from hUSLFs using TRIzol reagent (Invitrogen, United States) and combined with reverse transcriptase with Prime Script RT Master Mix kit (Takara, Japan). Relative gene expression was quantified by quantitative real-time reverse transcription polymerase chain reaction (qRT-PCR) performed in Applied Biosystems StepOnePlus Real-time PCR system (Applied Biosystems), using Fast SYBR Green Master Mix (Applied Biosystems). qPCR was performed as follows: 20 s at 95°C; 40 cycles of 3 s at 95°C and 30 s at 60°C; and 60–95°C for the dissociation curve. Relative gene expression was analyzed with 2^−ΔΔCt^ method and normalized relative to glyceraldehyde 3-phosphate dehydrogenase (GAPDH). The sequences of primers used were listed in Table 1.

**TABLE 1:** Primer sequence details of the analyzed gene (F: forward primer, R: reverse Primer).

Gene	Sequence
Human_GAPDH_F	TCG​GAG​TCA​ACG​GAT​TTG​GT
Human_GAPDH_R	TTC​CCG​TTC​TCA​GCC​TTG​AC
Human_ PARP1 _F	AGC​GTG​TTT​CTA​GGT​CGT​GG
Human_ PARP1 _R	CAT​CAA​ACA​TGG​GCG​ACT​GC
Human_ ERα_F	GGG​AAG​TAT​GGC​TAT​GGA​ATC​TG
Human_ ERα_R	TGG​CTG​GAC​ACA​TAT​AGT​CGT​T
Human_ ERβ_F	TTC​AAA​GAG​GGA​TGC​TCA​CTT​C
Human_ ERβ_R	CCT​TCA​CAC​GAC​CAG​ACT​CC
Human_ Bcl2_F	AGG​CTG​GGA​TGC​CTT​TGT​GGA​A
Human_ Bcl2_R	CAA​GCT​CCC​ACC​AGG​GCC​AAA
Human_ Bax_F	CCT​GTG​CAC​CAA​GGT​GCC​GGA​ACT
Human_ Bax_R	CCA​CCC​TGG​TCT​TGG​ATC​CAG​CCC
Human_ Ki67_F	GAG​GTG​TGC​AGA​AAA​TCC​AAA
Human_ Ki67_R	CTG​TCC​CTA​TGA​CTT​CTG​GTT​GT

### Determination of apoptotic cell distribution by flow cytometry

Cell apoptotic rate was examined using an PE Annexin V Apoptosis Detection Kit according to the manufacturer’s protocol (BD, United States). Briefly, cells from different groups were digested with trypsin (Gibco, United States), washed twice in cold DPBS, and re-suspended in 100 µl binding buffer. 5 μl PE Annexin V and 5 µl 7-AAD were added, followed by incubation for 15 min in the dark at room temperature. After staining, 300 μl binding buffer was added, and the cells were immediately analyzed by flow cytometry (BD, United States). The data were analyzed with FlowJo software.

### Western blot analysis

Effect of 17 β-estradiol on the expression of apoptosis-related molecules such as Bcl-2, cleaved PARP1, and PARP1 and estrogen receptor α and β was assessed by Western blot analysis. The cytoplasmic proteins were extracted from the cells by using lysis buffer. Cytoplasmic preparation was loaded into SDS-PAGE and electrophoresed under denaturing conditions. Subsequently, proteins were electro-transferred onto polyvinylidene fluoride transfer membrane. After blocking with 5% nonfat milk for 1 h, blots were incubated with primary antibodies such as ERα (1:1,000, Abcam, United States), ERβ (1:1,000, abclonal, China), Bcl-2 (1:1,000, CST, United States), PARP1 (1:1,000, CST, United States), cleaved PARP1 (1:1,000, CST, United States), or Tublin (1:5,000, CST, United States) antibodies for overnight followed by incubation with horseradish peroxidase-conjugated anti-mouse or anti-rabbit immunoglobulin (CST, United States) for 60 min. Visualization was achieved by using chemiluminescence reagents.

### Statistical analysis

Statistical analyses were performed with GraphPad Prism 8.0 (GraphPad, United States). The normally distributed clinical data are presented as means ± SD. Data that were not normally distributed, as determined using Kolmogorox-Smirnov test, were expressed as median with interquartile range. All the experiments were repeated at least three times. Student’s t-test or one-way analysis of variance (ANOVA) followed by Tukey’s post hoc testing, was used for comparisons between POP and control patients and multiple-group comparisons. *p* < 0.05 was considered statistically significant.

## Results

### Primary culture and identification of human uterosacral ligament fibroblasts

**FIGURE 1:**
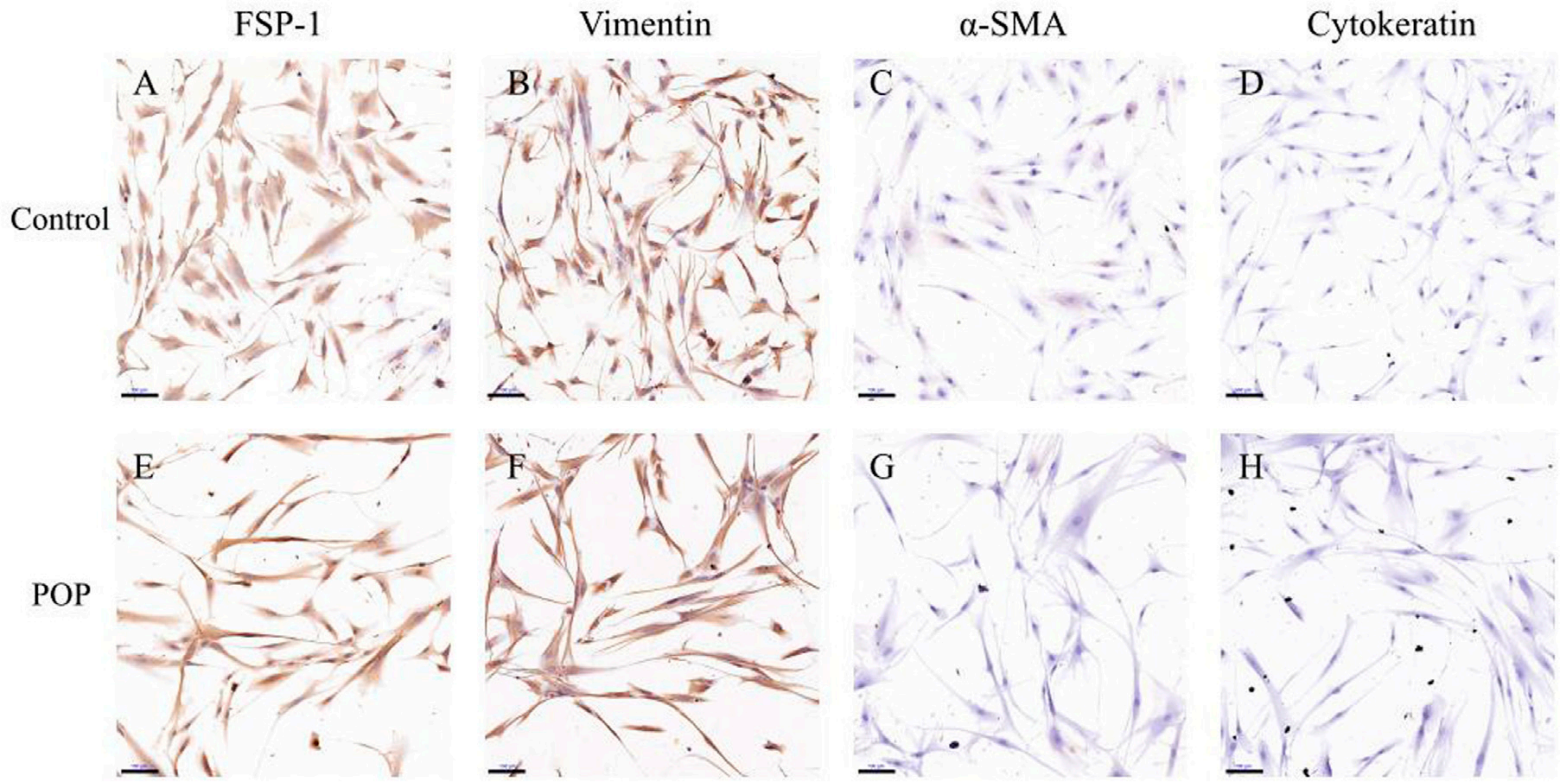
In the immunohistochemical staining of hUSLFs from Control **(A–D)** and POP **(E–H)** tissues, cells stained positive for FSP-1 **(A,E)** and vimentin **(B,F)**, and negative for α-SMA **(C,G)** and cytokeratin 5/6 **(D,H)**. Scale Bar = 100 μm.

In this study, we recruited five women with POP of POP-Q stages III and IV and five women without prolapse (control). The clinical characteristics including age, BMI, post menopause years and history of pregnancy and childbirth were recorded in Table 2. No significant differences in age, BMI, menopausal status, gravidity or vaginal parity were observed, indicating these characteristics were matched between the two groups. The fibroblasts mainly appeared as long spindles, but also as stellate shape as observed by light microscopy. Cells were connected to each other to form a network structure. Immunohistochemical staining showed that cells were positive for vimentin and FSP-1 (Figures 1A,B,E,F) and negative for cytokeratin and α-SMA (Figures 1C,D,G,H). Therefore, more than 90% of the cultured primary cells were hUSLFs, not smooth muscle cells or epithelial cells. Microscopically, no difference in morphology was observed between POP and control fibroblast cells.

**TABLE 2:** Clinical characteristics of enrolled patients.

Characteristic	Control (n = 5)	POP (n = 5)	*p*
Age(years)	54.80 ± 2.27	59.2 ± 3.84	0.39
BMI(kg/m^2^)	24.41 ± 0.59	23.82 ± 0.62	0.54
Proportion of post menopause	80.0%	80%	1.00
Gravidity	2.20 ± 0.37	3.2 ± 0.40	0.14
Parity	1.80 ± 0.49	1.60 ± 0.30	0.76

### Effect of 17 β-estradiol on cell proliferation in pelvic organ prolapse and control human uterosacral ligament fibroblasts

**FIGURE 2:**
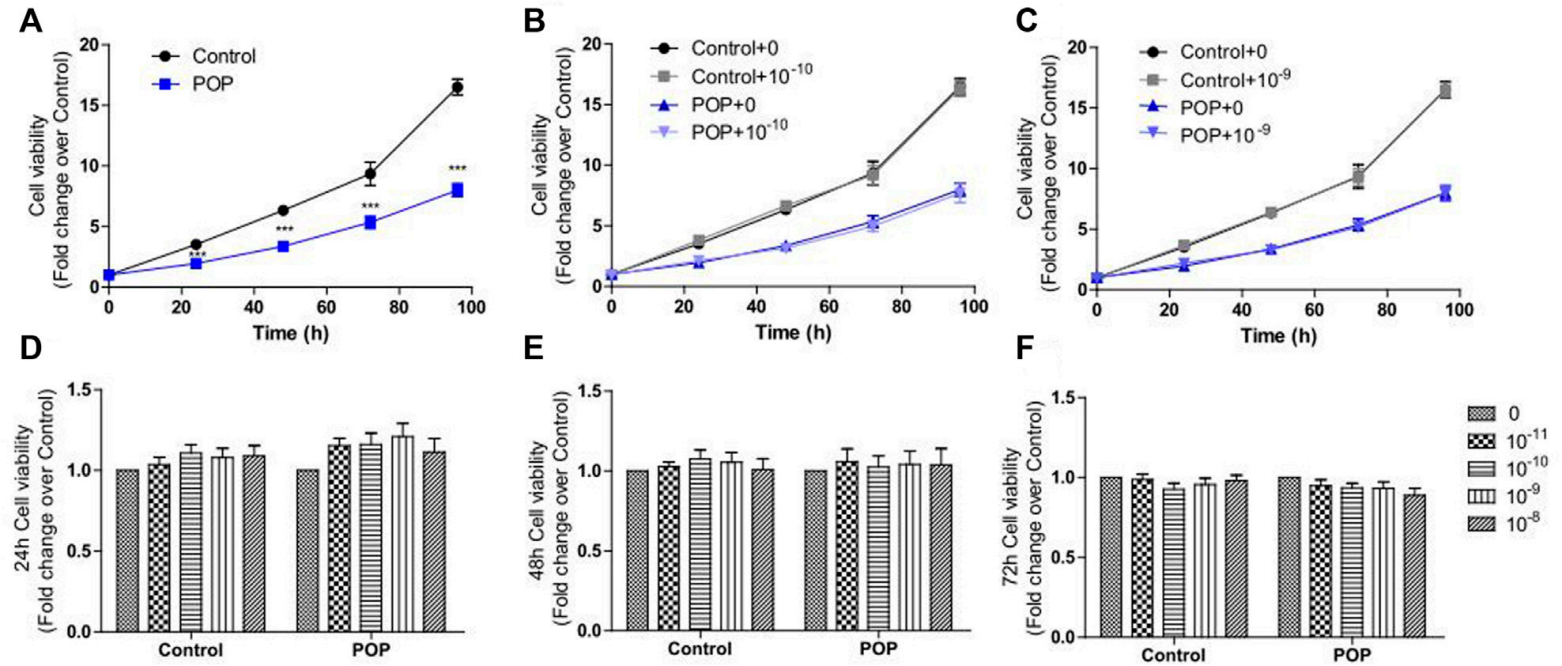
CCK8 assay was performed to determine the effects of 17 β-estradiol on cell proliferation in hUSLFs. The fibroblast cell growth rate of POP was obviously lower than that of Control **(A)**. Physiological 17 β-estradiol concentration (10^−10^ M**(B)** and 10^−9^ M**(C)** did not obviously influence the cell proliferation for both groups in the entire culture process. Estrogen treatment increased the proliferation of both control and POP fibroblast cells at 24 h, but the increase was not statistically significant **(D)** and did not continue in 48 h **(E)** and 72 h **(F)**. Values are expressed as the mean ± standard deviation of three independent experiments. ***p < 0.001 POP vs Control.

The fibroblasts growth rate of POP patients was significantly lower than controls shown in the CC8 assay (Figure 2A). Then both fibroblasts were treated with different concentrations of 17 β-estradiol. In this dose-response study, both patient and control fibroblasts showed slightly increased cell proliferation only at time point of 24 h (Figure 2D). Moreover, at physiological 17 β-estradiol concentration (10^−10^ and 10^−9^ M), both patient and control fibroblasts showed an increase in cell viability in comparison with other concentrations (Figures 2B,C). However, the enhancement was not statistically significant and did not continue in the following time points after 24 h as shown in Figures 2E,F. Meanwhile, fibroblasts derived from patients and controls showed no difference in proliferative rate under various 17 β-estradiol concentrations measured.

### Mechanical stress loading induces the cell death of fibroblasts

**FIGURE 3:**
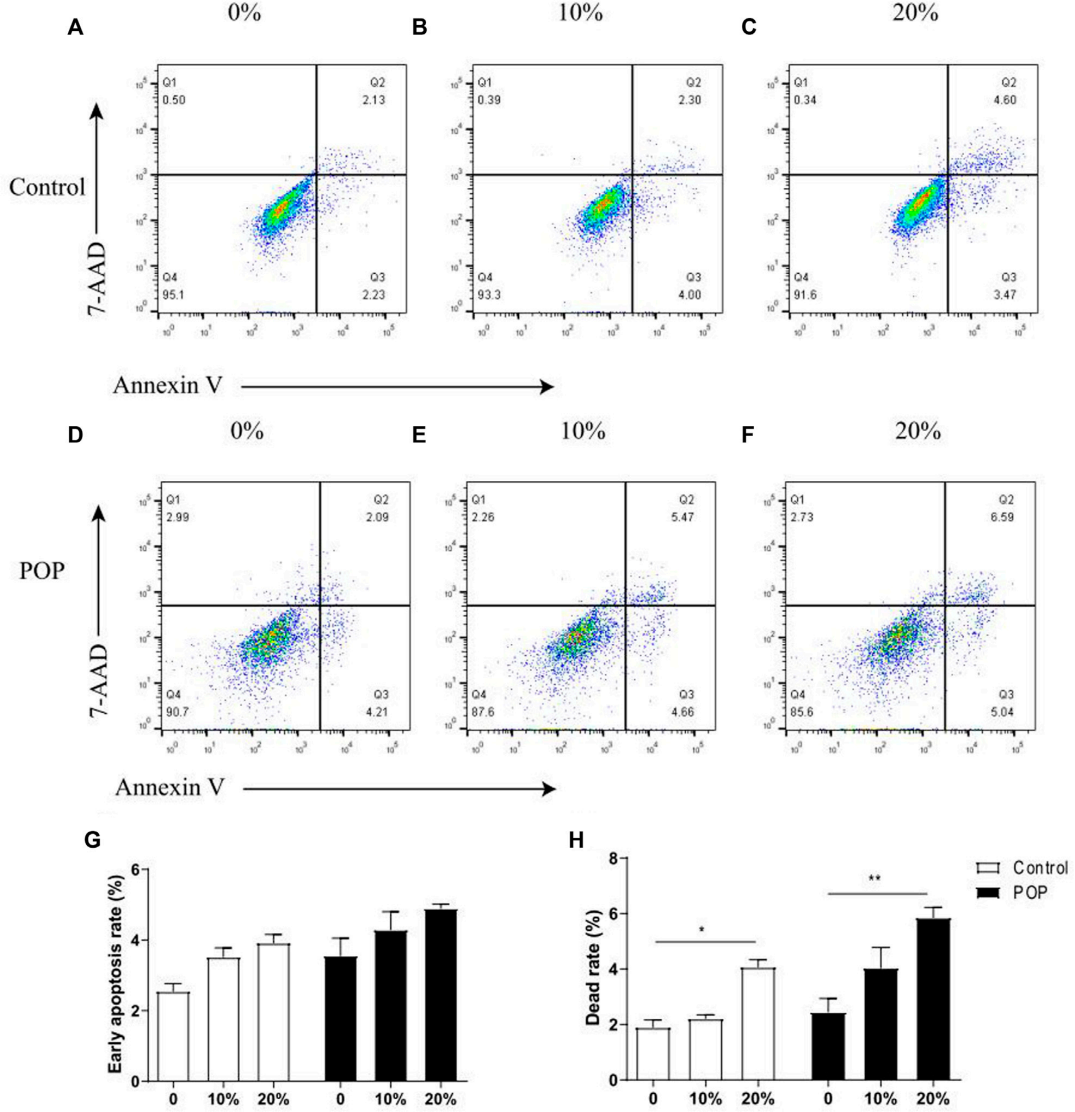
Mechanical stress induced apoptosis of hUSLFs occurs in a loading-dependent manner. **(A)** Representative dot plots of cell apoptosis by flow cytometry analysis after Annexin V/7-AAD dual staining. The apoptotic rate was determined as the percentage of 7-AAD negative and Annexin V positive (Q3). Cells that are already dead are both Annexin V and 7-AAD positive (Q2). **(A,D)** control; **(B,E)** 10% strain, 0.5 Hz; **(C,F)** 20% strain, 0.5 Hz. **(G)** Quantified apoptotic rates of hUSLFs treated with various intensities of mechanical strains for 24 h. **(H)** Quantified dead rates of hUSLFs treated with various intensities of mechanical strains for 24 h. Values are expressed as the mean ± standard deviation of three independent experiments. *p < 0.05; ***p < 0.001 vs. control.

The quantified results showed that a tensile strain of 10% did not markedly affect the early apoptosis and cell death rate (Figures 3B,E), while strains of 20% (Figures 3C,F) significantly increased the cell death rate compared with the group with no tensile strain (Figures 3A,D). Flow cytometric analysis showed the percentage of cells in early apoptosis with Annexin V positive and 7-AAD negative was elevated with the increasing tensile strain, and there was no difference between POP and control fibroblasts (Figure 3G). However, the percentage of annexin V positive and 7-AAD positive dead cells was significantly increased after stimulation with a tensile strain of 20% mechanical stretch compared with 0% mechanical stretch (Figure 3H), and the dead cell rate was a little bit higher in POP than control under the 20% mechanical stretch.

### 17 β-estradiol improves the mechanical stress induced cell apoptosis and death

**FIGURE 4:**
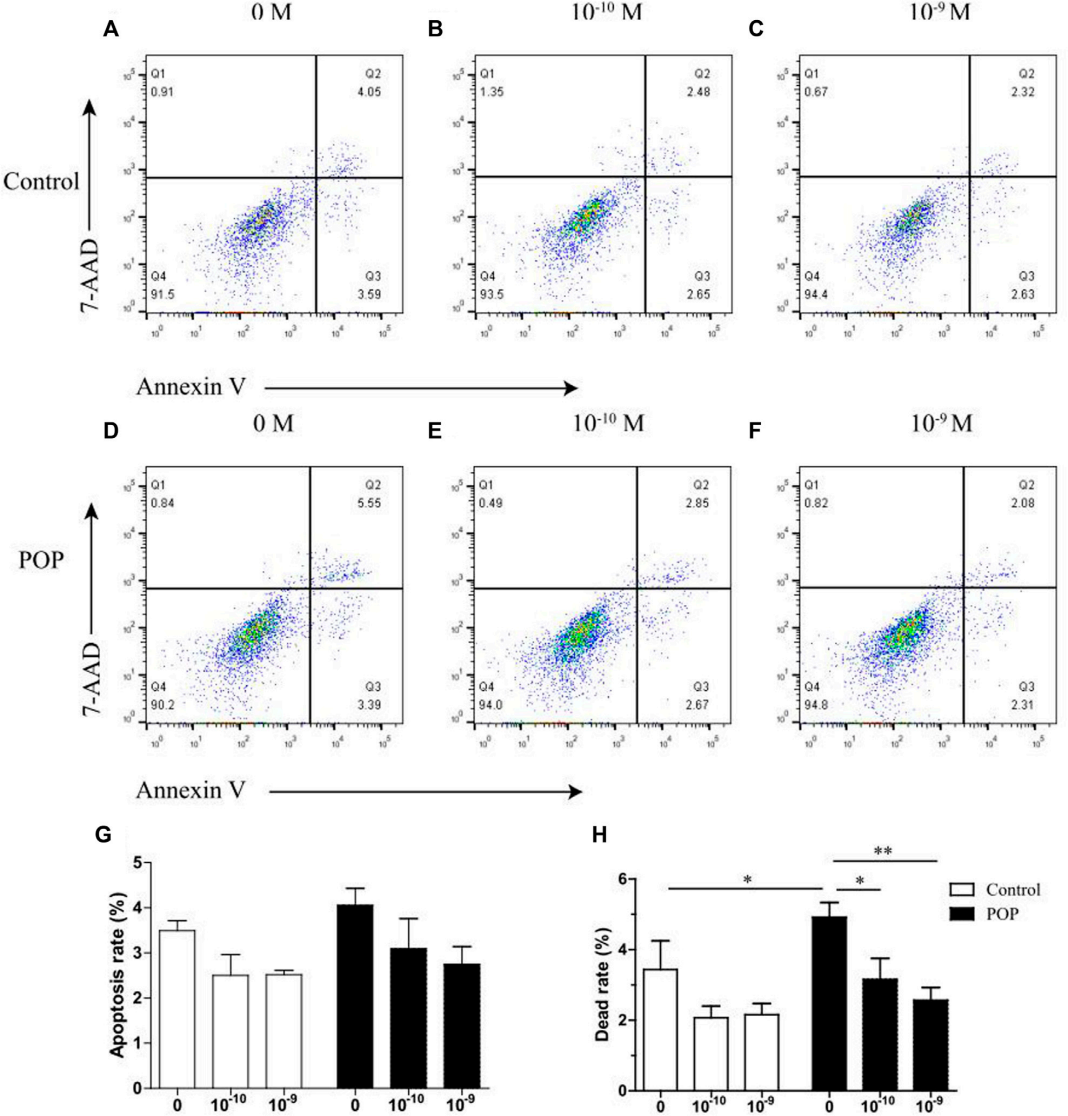
Effect of estrogen on mechanical stress induced apoptosis of hUSLFs occurs in a dose-dependent manner. **(A)** Representative dot plots of cell apoptosis by flow cytometry analysis after Annexin V/7-AAD dual staining. The apoptotic rate was determined as the percentage of 7-AAD negative and Annexin V positive (Q3). Cells that are already dead are both Annexin V and 7-AAD positive (Q2). **(A,D)** 0 M estrogen treatment; **(B,E)** 10^−10^ M estrogen treatment; (C and F) 10^−9^ M estrogen treatment. **(G)** Quantified apoptotic rates of hUSLFs treated with various concentrations of estrogen for 24 h, 0.5 Hz, 20% strain. **(H)** Quantified dead rates of hUSLFs treated with various concentrations of estrogen for 24 h, 0.5 Hz, 20% strain. Values are expressed as the mean ± standard deviation of three independent experiments. *p < 0.05; **p < 0.01 vs. control.

Results showed that estrogen improved the cell viabiltiy impared by machenical stimulation in two groups. (Figures 4A–F). The apoptosis rate increased by the estrogen is not statistical significant. In particularly, the induction of dead cells by mechanical strain could be reversed by 17 β-estradiol in POP group, but this effect was not significant in control group which is due to the relatively mild cell death caused by mechanical stress (Figure 4H).

### 17 β-estradiol reduces the mechanical strain-induced cell death by regulating poly-ADP-ribose polymerase pathway

**FIGURE 5:**
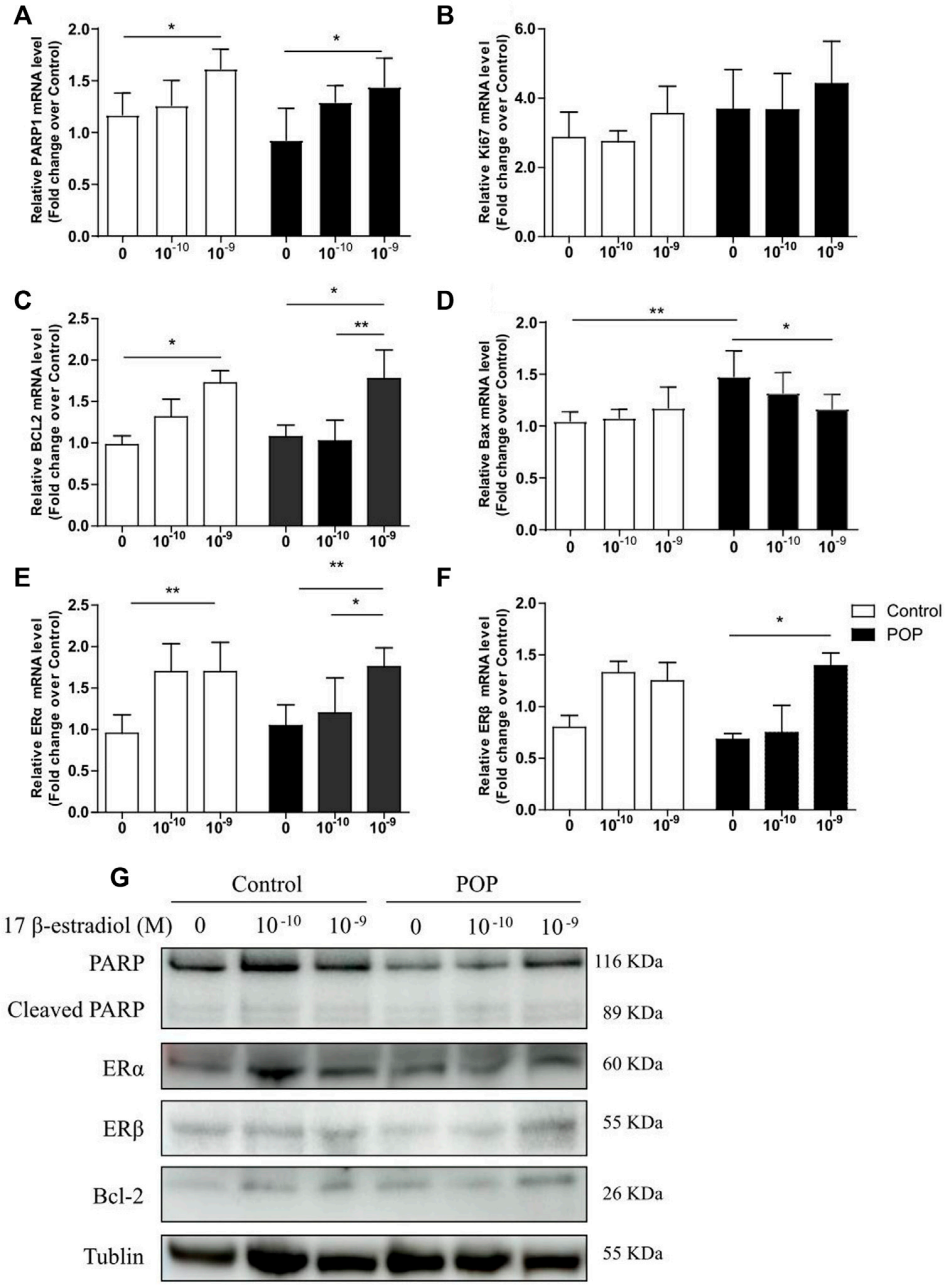
Estrogen regulates apoptosis-related proteins expression in hUSLFs. mRNA expression levels of apoptosis-related genes including PARP1, Ki67, BCL2, Bax, ERα, and ERβ in hUSLFs **(A–F)**. Protein expression of PARP1, Cleaved-PARP1, ERα, ERβ, BCL2 and Tublin in hUSLFs **(G)**. Values are expressed as the mean ± standard deviation of three independent experiments. *p < 0.05; **p < 0.01.

To clarify the mechanism of estrogen-reversed apoptosis, which was induced by the mechanical strain, the mRNA and protein levels of anti- and pro-apoptotic proteins following estrogen treatment were examined by qRT-PCR and immunoblotting. Estrogen treatment increased mRNA abundance of PARP1 (poly-ADP-ribose polymerase), Ki67 and Bcl-2 in two groups (Figures 5A–C). Figure 5G showed that the PARP1 and Bcl-2 protein levels gradually increased when treated with 17 β-estradiol in both control and POP cells. Of these, the PARP1 expression under different 17 β-estradiol concentrations were lower in POP cells when compared to Control. However, no significant difference was found in the cleaved PARP1 levels between the two group after treated with 17 β-estradiol. Meanwhile, 17 β-estradiol slightly decreased the mRNA expression of Bax in POP, which were not observed in control cells (Figure 5D). Considering PARP1 as a key regulator of ERα in controlling ERα transactivation (Zhai et al., 2015), the mRNA and protein levels of ERα and ERβ was also measured and displayed the similar trend like PARP1, which grew with the estrogen concentration (Figures 5E,F,G). Furthermore, ERα was the major estrogen receptors subtype in hUSLFs (Figure 5G). The results collectively raised the possibility that estrogen exhibits anti-apoptosis activities through induction of PARP1 in hUSLFs under mechanical stress. Further studies are needed to examine the specific mechanism of PARP pathway induced by estrogen.

## Discussion

POP is not life-threatening but has a significant impact on a patient’s quality of life with its incidence increasing every year. It is well accepted that POP is associated with postmenopausal estrogen deficiency and overstretching of the pelvic floor lead by vaginal delivery (Deng et al., 2021). The current study found that overall growth rate of POP fibroblasts was significantly slower than controls under culture conditions, and estrogen slightly increased cell proliferation of all the fibroblasts with the most prominent effect at physiological concentrations (10^−10^ and 10^−9^ M), and the enhancement was only observed at 24 h, perhaps because of the half-life of estrogen is about 24 h in the culture medium. Similar results were also confirmed in a study assessing the growth rate of fibroblasts from patients with POP and controls with 3-(4, 5, -dimethyl thiazolyl-2) -2, 5-diphenyl tetrazolium bromide (MTT) assay (Liu et al., 2006). The possible cause for the inconsistence of estrogen effect on fibroblast cells can be that the sample size is relatively too small in the 2006 (seven per group) and this study (five per group) to get uniform conclusions. Secondly, the big mean age difference (15 years old) of POP and controls may somewhat explain the inhibition effect of estrogen in POP patients. In this study, CCK8 assay was used to examine the cell viability which is better than MTT assay in the aspects of detection sensitivity, accuracy, repeatability and cytotoxicity (Liu, 2013). Alteration in cell growth may affect the production of collagen, elastic fibres and ECMs and the associated synthesis or turnover, assembly, cross-linking and remodeling. Actually, in our previous study, decrease of collagens (COLI and COLIII) and increase of Bax in mRNA levels was found in POP hUSLFs (Zhu et al., 2021). Pelvic floor relies on the pelvic floor fascia and ligaments for its support, and the main component of the ligaments is collagen. Thus, exogenous 17 β-estradiol might indirectly improve the collagen formation for uterosacral ligament, but this effect is not statistically significant due to its limited action time or half-life.

In addition, the death rate of fibroblasts subjected to 20% mechanical strain was significantly increased compared with that in fibroblast with no intensity strain, especially in POP fibroblasts. Previous studies on the pathological mechanism of pelvic organ prolapse focused on cell apoptosis of pelvic supporting structures (Wen et al., 2011; Hong et al., 2015b), which showed that mechanical strain enhanced intracellular ROS levels, decreased the mitochondrial membrane potential. Meanwhile, 20% mechanical strain induced cell apoptosis was reversed by estrogen treatment in both groups. Our previous proteomic analysis indicated that many apoptosis-related proteins were differentially expressed in the uterosacral ligaments of POP patients (Sun et al., 2015). Thus apoptosis-related proteins were mainly measured in hUSLFs and it was confirmed that the anti-apoptotic Bcl-2 were enhanced and pro-apoptotic Bax was weaken by estrogen administration under the mechanical load condition. It is worth noting that both mRNA and protein expression of PARP1 was stimulated by estrogen in hUSLFs. PARP1 is a ubiquitous multifunctional nuclear enzyme that catalyzes the transfer of ADP-ribose units from NAD^+^ to specific target proteins and controls important physiological processes such as DNA methylation, DNA damage response, chromatin remodeling and gene expression (Srivastava and Lodhi, 2022) (Swindall et al., 2013) (Hassa and Hottiger, 2008). Several studies have proved that ERα could be poly(ADP-ribosyl)ated by binding to PARP1 and promoting ERα-dependent gene transcription (Zhang et al., 2013; Gadad et al., 2021). In premenopausal women with POP, serum estrogen levels and ERα values measured by immunohistochemical staining in the cardinal and uterosacral ligaments were significantly decreased as compared to women without POP (Lang et al., 2003). Estrogen, the ligand of ERα, not only increased the ERα expression, but also enhanced PARP1 expression, especially in POP hUSLFs. The PARP1 inhibitors have been exploited clinically for the treatment of various cancers (Ray Chaudhuri and Nussenzweig, 2017). Therefore, a comprehensive understanding of the crucial roles of PARP1 in DNA repair is of paramount importance for the protective effect of exogenous estrogen on the cell viability of uterosacral ligaments fibroblast.

Our study proved the impaired proliferate capacity in POP fibroblasts and the ability of estrogen to ameliorate mechanical stress induced cell apoptosis and death on fibroblast *in vitro,* especially in hUSLFs from POP patients. In postmenopausal women, serum estrogen decreases rapidly and the connective tissue cannot be repaired effectively, resulting in a reduction in the number of fibroblasts, loss of elasticity, and decreased posttranslational processing of collagen (Calleja-Agius and Brincat, 2012). Our finding suggested that estrogen defends the muscles and fascia of the pelvic floor against the damage caused by mechanical strain such as vaginal delivery, but the protective effect diminished in postmenopausal women due to the rapid reduction of estrogen levels, which to some extent explains why POP mainly occurs after menopause. Recently, estrogen was proved to restrain the progress of POP by inhibiting the expression level of Mitofusin-2 and promoting expression of procollagens and proliferation of fibroblasts (Wang et al., 2020). Moreover, estrogen is known to have a positive effect on epithelial thickness and blood flow (Trutnovsky, 2021), and therefore is well accepted for prolapse-associated complications therapy. However, application of estrogen to facilitate prolapse-associated symptoms in postmenopausal women with symptomatic pelvic organ prolapse remains controversial. Other reports showed that preoperative local estrogen cream does not ameliorate prolapse-associated symptoms in postmenopausal women with pelvic organ prolapse (Marschalek et al., 2021). However, a 30-day oral estrogen therapy in postmenopausal women with or without prolapse found that hyaluronic acid, a predominant component of the ECM-sulfated glycosaminoglycan-in the parametrium and vaginal apex of women, was increased in the parametrium of women receiving estrogen compared to those treated with the placebo (Nunes et al., 2011). It seems that the estrogen delivery way affects the therapeutic efficacy and oral administration is better than vaginal estrogen cream.

In conclusion, we found that cell proliferation of uterosacral ligament fibroblasts from patients with POP is lower than controls. The results indicated that estrogen seems to exhibit the protective effect only when cell was challenged with mechanical stain through the stimulation of PARP expression. In this view, we propose that rigorous and more comprehensive studies are needed on the role of exogenous estrogen administration as a form of POP prevention.

## Data Availability

The original contributions presented in the study are included in the article/Supplementary Material, further inquiries can be directed to the corresponding author.

## Abbreviations

BMI, body mass index; CCK8, Cell counting Kit 8; DPBS, Dulbecco’s phosphate-buffered saline; DMEM, Dulbecco’s modified Eagle’s medium; ECM, extracellular matrix; FSP-1, fibroblast specific protein 1; APDH, glyceraldehyde 3-phosphate dehydrogenase; hUSLFs, human uterosacral ligament fibroblasts; PARP1, poly-ADP-ribose polymerase; POP, pelvic organ prolapses; qRT-PCR, Quantitative real-time polymerase chain reaction; SERM, selective estrogen receptor modulators; 7-AAD, 7-Amino-Actinomycin.

## References

[B1] Al-Azzawi F. Ewies A. Thompson J. (2003). Changes in extracellular matrix proteins in the cardinal ligaments of post-menopausal women with or without prolapse: A computerized immunohistomorphometric analysis. Hum. Reprod. 18, 2189–2195. 10.1093/humrep/deg420 14507843

[B2] Calleja-Agius J. Brincat M. (2012). The effect of menopause on the skin and other connective tissues. Gynecol. Endocrinol. 28 (4), 273–277. 10.3109/09513590.2011.613970 21970508

[B3] Clark A. L. Slayden O. D. Hettrich K. Brenner R. M. (2005). Estrogen increases collagen I and III mRNA expression in the pelvic support tissues of the rhesus macaque. Am. J. Obstet. Gynecol. 192 (5), 1523–1529. 10.1016/j.ajog.2004.11.042 15902152

[B4] Deng Z-M. Dai F-F. Yuan M-Q. Yang D-Y. Zheng Y-J. Cheng Y-X. (2021). Advances in molecular mechanisms of pelvic organ prolapse (Review). Exp. Ther. Med. 22 (3), 1009. 10.3892/etm.2021.10442 34345291 PMC8311251

[B5] Gadad S. S. Camacho C. V. Malladi V. Hutti C. R. Nagari A. Kraus W. L. (2021). PARP-1 regulates estrogen-dependent gene expression in estrogen receptor α–positive breast cancer cells. Mol. Cancer Res. 19 (10), 1688–1698. 10.1158/1541-7786.MCR-21-0103 34158394 PMC8492518

[B6] Goldstein S. R. Nanavati N. (2002). Adverse events that are associated with the selective estrogen receptor modulator levormeloxifene in an aborted phase III osteoporosis treatment study. Am. J. Obstet. Gynecol. 187 (3), 521–527. 10.1067/mob.2002.123938 12237621

[B7] Hassa P. O. Hottiger M. O. (2008). The diverse biological roles of mammalian PARPS, a small but powerful family of poly-ADP-ribose polymerases. Front. Biosci. 13, 3046–3082. 10.2741/2909 17981777

[B8] Hong S. Hong L. Wu D. Li B. Liu C. Guo W. (2015). Oxidative damage to human parametrial ligament fibroblasts induced by mechanical stress. Mol. Med. Rep. 12 (4), 5342–5348. 10.3892/mmr.2015.4115 26238938

[B9] Hong S. Li H. Wu D. Li B. Liu C. Guo W. (2015). Oxidative damage to human parametrial ligament fibroblasts induced by mechanical stress. Mol. Med. Rep. 12, 5342–5348. 10.3892/mmr.2015.4115 26238938

[B10] Lang J. H. Zhu L. Sun Z. J. Chen J. (2003). Estrogen levels and estrogen receptors in patients with stress urinary incontinence and pelvic organ prolapse. Int. J. Gynaecol. Obstet. 80 (1), 35–39. 10.1016/s0020-7292(02)00232-1 12527458

[B11] Liu A. Q. (2013). Comparative study on the optimal experiment conditions between CCK-8 and MTT in rabbit fibroblasts. Beijing, China: Medical Innovation of China.

[B12] Liu Y. Choy K. W. Lui W. T. Pang M. W. Wong Y. F. Yip S. K. (2006). 17^2^-Estradiol suppresses proliferation of fibroblasts derived from cardinal ligaments in patients with or without pelvic organ prolapse. Hum. Reprod. 21, 303–308. 10.1093/humrep/dei296 16155073

[B13] Marschalek M. L. Bodner K. Kimberger O. Zehetmayer S. Morgenbesser R. Dietrich W. (2021). Does preoperative locally applied estrogen treatment facilitate prolapse-associated symptoms in postmenopausal women with symptomatic pelvic organ prolapse? A randomised controlled double-masked, placebo-controlled, multicentre study. BJOG An Int. J. Obstetrics Gynaecol. 128 (13), 2200–2208. 10.1111/1471-0528.16894 PMC9293194 34464489

[B14] Ming H. Li H. Hong S. Jie M. Zhao Y. Yang Q. (2017). Mechanical stress influences the viability and morphology of human parametrial ligament fibroblasts. Mol. Med. Rep. 15 (2), 853–858. 10.3892/mmr.2016.6052 28000871

[B15] Nunes J. M. Feldner P. C. Jr. Castro R. A. Nader H. B. Sartori M. G. Girão M. J. (2011). Uterine prolapse: Evaluation of glycosaminoglycans in postmenopausal women after estrogen therapy. Climacteric 14 (1), 121–125. 10.3109/13697137.2010.500010 20690864

[B16] Pang H. Zhang L. Han S. Li Z. Gong J. Liu Q. (2021). A nationwide population-based survey on the prevalence and risk factors of symptomatic pelvic organ prolapse in adult women in China - a pelvic organ prolapse quantification system-based study. BJOG 128 (8), 1313–1323. 10.1111/1471-0528.16675 33619817 PMC8252658

[B17] Rahn D. D. Good M. M. Roshanravan S. M. Shi H. Schaffer J. I. Singh R. J. (2014). Effects of preoperative local estrogen in postmenopausal women with prolapse: A randomized trial. J. Clin. Endocrinol. Metab. 99 (10), 3728–3736. 10.1210/jc.2014-1216 24947034 PMC4184065

[B18] Ray Chaudhuri A. Nussenzweig A. (2017). The multifaceted roles of PARP1 in DNA repair and chromatin remodelling. Nat. Rev. Mol. Cell Biol. 18 (10), 610–621. 10.1038/nrm.2017.53 28676700 PMC6591728

[B19] Srivastava R. Lodhi N. (2022). DNA methylation malleability and dysregulation in cancer progression: Understanding the role of PARP1. Biomolecules 12 (3), 417. 10.3390/biom12030417 35327610 PMC8946700

[B20] Sun Z-J. Zhu L. Lang J-H. Wang Z. Liang S. (2015). Proteomic analysis of the uterosacral ligament in postmenopausal women with and without pelvic organ prolapse. Chin. Med. J. 128 (23), 3191–3196. 10.4103/0366-6999.170262 26612295 PMC4794882

[B21] Swindall A. F. Stanley J. A. Yang E. S. (2013). PARP-1: Friend or foe of DNA damage and repair in tumorigenesis? Cancers 5 (3), 943–958. 10.3390/cancers5030943 24202328 PMC3795373

[B22] Tinelli A. Malvasi A. Rahimi S. Negro R. Vergara D. Martignago R. (2010). Age-related pelvic floor modifications and prolapse risk factors in postmenopausal women. Menopause 17 (1), 204–212. 10.1097/gme.0b013e3181b0c2ae 19629013

[B23] Trutnovsky G. (2021). Vaginal estrogen is unlikely to improve advanced prolapse symptoms. BJOG An Int. J. Obstetrics Gynaecol. 128 (13), 2208. 10.1111/1471-0528.16900 34473892

[B24] Vergeldt T. F. M. Weemhoff M. IntHout J. Kluivers K. B. (2015). Risk factors for pelvic organ prolapse and its recurrence: A systematic review. Int. Urogynecol. J. 26 (11), 1559–1573. 10.1007/s00192-015-2695-8 25966804 PMC4611001

[B25] Wang S. Zhang Z. Lü D. Xu Q. (2015). Effects of mechanical stretching on the morphology and cytoskeleton of vaginal fibroblasts from women with pelvic organ prolapse. Int. J. Mol. Sci. 16 (5), 9406–9419. 10.3390/ijms16059406 25923074 PMC4463595

[B26] Wang X-Q. He R-J. Xiao B-B. Lu Y. (2020). Therapeutic effects of 17β-estradiol on pelvic organ prolapse by inhibiting Mfn2 expression: An *in vitro* study. Front. Endocrinol. 11, 586242. 10.3389/fendo.2020.586242 PMC7726108 33324344

[B27] Wasenda E. J. Kamisan Atan I. Subramaniam N. Dietz H. P. (2017). Pelvic organ prolapse: Does hormone therapy use matter? Menopause 24 (10), 1185–1189. 10.1097/GME.0000000000000898 28538602

[B28] Weintraub A. Y. Glinter H. Marcus-Braun N. (2020). Narrative review of the epidemiology, diagnosis and pathophysiology of pelvic organ prolapse. Int. Braz J. Urol. 46 (1), 5–14. 10.1590/S1677-5538.IBJU.2018.0581 31851453 PMC6968909

[B29] Wen Y. Ho J. Y-P. Polan M. L. Chen B. (2011). Expression of apoptotic factors in vaginal tissues from women with urogenital prolapse. Neurourol. Urodyn. 30 (8), 1627–1632. 10.1002/nau.21127 21674599 PMC4507512

[B30] Zhai L. Li S. Li H. Zheng Y. Lang R. Fan Y. (2015). Polymorphisms in poly (ADP-ribose) polymerase-1 (PARP1) promoter and 3' untranslated region and their association with PARP1 expression in breast cancer patients. Int. J. Clin. Exp. Pathol. 8 (6), 7059–7071. Available from: http://europepmc.org/abstract/MED/26261599 . 26261599 PMC4525933

[B31] Zhang F. Wang Y. Wang L. Luo X. Huang K. Wang C. (2013). Poly(ADP-ribose) polymerase 1 is a key regulator of estrogen receptor α-dependent gene transcription. J. Biol. Chem. 288 (16), 11348–11357. 10.1074/jbc.M112.429134 23493398 PMC3630903

[B32] Zhu Y. Li L. Xie T. Guo T. Zhu L. Sun Z. (2021). Mechanical stress influences the morphology and function of human uterosacral ligament fibroblasts and activates the p38 MAPK pathway. Int. Urogynecol. J. 33, 2203–2212. 10.1007/s00192-021-04850-7 34036402 PMC9343297

